# PHOG-BLAST – a new generation tool for fast similarity search of protein families

**DOI:** 10.1186/1471-2148-6-51

**Published:** 2006-06-22

**Authors:** Igor V Merkeev, Andrey A Mironov

**Affiliations:** 1 State Scientific Center GosNIIGenetica, 1st Dorozhny pr., 1, Moscow, 113545, Russia; 2 Department of Bioengineering and Bioinformatics, Moscow State University, Vorob'evy gory, 1–73, Moscow, 119992, Russia

## Abstract

**Background:**

The need to compare protein profiles frequently arises in various protein research areas: comparison of protein families, domain searches, resolution of orthology and paralogy. The existing fast algorithms can only compare a protein sequence with a protein sequence and a profile with a sequence. Algorithms to compare profiles use dynamic programming and complex scoring functions.

**Results:**

We developed a new algorithm called PHOG-BLAST for fast similarity search of profiles. This algorithm uses profile discretization to convert a profile to a finite alphabet and utilizes hashing for fast search. To determine the optimal alphabet, we analyzed columns in reliable multiple alignments and obtained column clusters in the 20-dimensional profile space by applying a special clustering procedure. We show that the clustering procedure works best if its parameters are chosen so that 20 profile clusters are obtained which can be interpreted as ancestral amino acid residues. With these clusters, only less than 2% of columns in multiple alignments are out of clusters. We tested the performance of PHOG-BLAST vs. PSI-BLAST on three well-known databases of multiple alignments: COG, PFAM and BALIBASE. On the COG database both algorithms showed the same performance, on PFAM and BALIBASE PHOG-BLAST was much superior to PSI-BLAST. PHOG-BLAST required 10–20 times less computer memory and computation time than PSI-BLAST.

**Conclusion:**

Since PHOG-BLAST can compare multiple alignments of protein families, it can be used in different areas of comparative proteomics and protein evolution. For example, PHOG-BLAST helped to build the PHOG database of phylogenetic orthologous groups. An essential step in building this database was comparing protein complements of different species and orthologous groups of different taxons on a personal computer in reasonable time. When it is applied to detect weak similarity between protein families, PHOG-BLAST is less precise than rigorous profile-profile comparison method, though it runs much faster and can be used as a hit pre-selecting tool.

## Background

The availability of many completely sequenced genomes provides rich material for studying protein evolution. Gene duplications, gene losses, gene acquisitions and horizontal transfer of genes make it very difficult to reconstruct the exact evolutionary history of a protein family. A widely used approach to study such history is to find orthologs and paralogs by comparing completely sequenced genomes. Orthologs are genes derived from a single ancestral gene as a result of the speciation event, while paralogs are genes that result from gene duplication events [1-3]. How can we resolve these evolutionary relationships for hundreds of organisms having thousands of genes using just an ordinary personal computer? One possible approach is to create orthologous groups for each node of the evolutionary tree [4] and to compare orthologous groups belonging to different nodes of the tree. Since each orthologous group is represented by a multiple alignment of protein sequences, we need a very fast algorithm to compare multiple alignments.

Profiles represent a very sensitive technique to represent a protein family and to compare protein families. They were introduced by Michael Gribskov and co-workers [5] and they have proved to be a valuable tool for finding weak similarities between distant proteins belonging to one family or superfamily and for improving sensitivity of database searches [6,7]. Two views have been established on the composition of profiles: continuous and discrete. The continuous model of profiles imposes no restriction on amino acids and their counts present in an alignment column, while the discrete model assumes that only certain amino acid residues can be found at a certain position in the multiple alignment of a protein family.

The efforts of several authors were directed to improve profiles by sequence weighting [8-12] and by introducing pseudocounts [13-16]. As it was pointed by Roman Tatusov and co-workers [6], the most efficient method in improving the quality of profiles by adding pseudocounts is the Dirichlet mixture. This mixture is a linear combination of Dirichlet distributions. Although no restrictions are imposed on frequency profiles in this model, Dirichlet distributions can be considered as discrete condensation points in 20-dimensional space.

The discrete view on profiles is exemplified by the PROSITE database [17], which states what kind of amino acids can be present in a single position of a protein signature.

Existing procedures for profile-profile comparisons [18-22] use dynamic programming and complex scoring functions between profiles like dot product to score a column from one profile aligned to a column of the other profile. These methods were successfully used to detect weak similarities between different protein families, to recognize folds and to predict 3-D structure. A pairwise alignment of profiles using dynamic programming requires A^2^*m*n operations, where A is the alphabet size (for proteins A = 20), m and n are protein lengths. Fast heuristic BLAST-like procedures require a discrete alphabet and it is not possible to use them to compare a pair of profiles. If we are able somehow to convert profiles to discrete values, then we can substitute an alignment column with a symbol denoting the column type. To this end, all alignment columns from the BLOCKS database were converted to frequency distributions and a special clustering procedure was applied to them. It appeared that more than 98% of alignment columns in multiple alignments from standard databases of protein multiple alignments belong to just 20 clusters, each with its own dominating amino acid residue ("ancestral" residue). Therefore, it is possible to substitute an alignment column with this dominating residue that can be also thought as the "ancestral" amino acid residue from which the alignment column evolved. In the case we cannot assign an alignment column to its "ancestral" residue, a special symbol "X" is used, denoting a "garbage" column.

When "ancestral" sequences are obtained it is possible to find a similarity score by applying the rigorous Smith-Waterman algorithm [23]. But it is more desirable to develop an algorithm that might be somewhat not so rigorous but fast enough to allow a researcher to perform very computationally intensive tasks, for example, to compare orthologous groups of different taxons on a personal computer in reasonable time.

## Implementation

### Clustering procedure

Each alignment column from a database of multiple alignments was converted to the frequency vector using a simple formula:

f→=(f1,f2,…,f20)fi=NiN
 MathType@MTEF@5@5@+=feaafiart1ev1aaatCvAUfKttLearuWrP9MDH5MBPbIqV92AaeXatLxBI9gBaebbnrfifHhDYfgasaacH8akY=wiFfYdH8Gipec8Eeeu0xXdbba9frFj0=OqFfea0dXdd9vqai=hGuQ8kuc9pgc9s8qqaq=dirpe0xb9q8qiLsFr0=vr0=vr0dc8meaabaqaciaacaGaaeqabaqabeGadaaakeaafaqadeGabaaabaGafmOzayMbaSaacqGH9aqpcqGGOaakcqWGMbGzdaWgaaWcbaGaeGymaedabeaakiabcYcaSiabdAgaMnaaBaaaleaacqaIYaGmaeqaaOGaeiilaWIaeSOjGSKaeiilaWIaemOzay2aaSbaaSqaaiabikdaYiabicdaWaqabaGccqGGPaqkaeaacqWGMbGzdaWgaaWcbaGaemyAaKgabeaakiabg2da9maalaaabaGaemOta40aaSbaaSqaaiabdMgaPbqabaaakeaacqWGobGtaaaaaaaa@44D6@

where *N*_*i *_is the number of times amino acid *i *occurs in the column, *N *is the number of sequences in the multiple alignment. Thus, we obtained the data set *D *of all frequency vectors computed from all alignments columns.

Following Shmuel Pietrokovski [24], we define similarity between column frequency vectors as the Pearson correlation coefficient *r*:

r(f→′,f→″)=∑i=120(f′i−f¯′)⋅(f″i−f¯″)∑i=120(f′i−f¯′)2⋅∑i=120(f″i−f¯″)2
 MathType@MTEF@5@5@+=feaafiart1ev1aaatCvAUfKttLearuWrP9MDH5MBPbIqV92AaeXatLxBI9gBaebbnrfifHhDYfgasaacH8akY=wiFfYdH8Gipec8Eeeu0xXdbba9frFj0=OqFfea0dXdd9vqai=hGuQ8kuc9pgc9s8qqaq=dirpe0xb9q8qiLsFr0=vr0=vr0dc8meaabaqaciaacaGaaeqabaqabeGadaaakeaacqWGYbGCcqGGOaakcuWGMbGzgaWcgaqbaiabcYcaSiqbdAgaMzaalyaagaGaeiykaKIaeyypa0ZaaSaaaeaadaaeWbqaaiabcIcaOiqbdAgaMzaafaWaaSbaaSqaaiabdMgaPbqabaGccqGHsislcuWGMbGzgaqegaqbaiabcMcaPiabgwSixlabcIcaOiqbdAgaMzaagaWaaSbaaSqaaiabdMgaPbqabaGccqGHsislcuWGMbGzgaqegaGbaiabcMcaPaWcbaGaemyAaKMaeyypa0JaeGymaedabaGaeGOmaiJaeGimaadaniabggHiLdaakeaadaGcaaqaamaaqahabaGaeiikaGIafmOzayMbauaadaWgaaWcbaGaemyAaKgabeaakiabgkHiTiqbdAgaMzaaryaafaGaeiykaKYaaWbaaSqabeaacqaIYaGmaaaabaGaemyAaKMaeyypa0JaeGymaedabaGaeGOmaiJaeGimaadaniabggHiLdGccqGHflY1daaeWbqaaiabcIcaOiqbdAgaMzaagaWaaSbaaSqaaiabdMgaPbqabaGccqGHsislcuWGMbGzgaqegaGbaiabcMcaPmaaCaaaleqabaGaeGOmaidaaaqaaiabdMgaPjabg2da9iabigdaXaqaaiabikdaYiabicdaWaqdcqGHris5aaWcbeaaaaaaaa@6E12@

We have chosen a variation of the classical "k-means" procedure [25] due to the simplicity of its implementation and easy analysis how it depends on its main parameter. Since we do now know the number of clusters in the data set *D*, we can setup a correlation coefficient threshold *r*_*thresh *_and then merge clusters *C*_*i *_and *C*_*j *_if the distance between them is less than *r*_*thresh*_. Our modified "k-means" procedure will run as follows.

1. For each frequency vector **x **find all frequency vectors **y **such that *r*(**x**,**y**)<*r*_*thresh*_. These frequency vectors will form an initial cluster. If a frequency vector is already included in an initial cluster, it is not considered as a seed for a new cluster. Compute the initial means *μ*_1_,...,*μ*_*k*_.

2. Assign each frequency vector **x **in *D *to the cluster *C*_*i *_whose mean *μ*_*i *_is the nearest to **x.**

3. Recompute the means of all the clusters.

μi=(1/|Ci|)∑x∈Cix
 MathType@MTEF@5@5@+=feaafiart1ev1aaatCvAUfKttLearuWrP9MDH5MBPbIqV92AaeXatLxBI9gBaebbnrfifHhDYfgasaacH8akY=wiFfYdH8Gipec8Eeeu0xXdbba9frFj0=OqFfea0dXdd9vqai=hGuQ8kuc9pgc9s8qqaq=dirpe0xb9q8qiLsFr0=vr0=vr0dc8meaabaqaciaacaGaaeqabaqabeGadaaakeaaiiGacqWF8oqBdaWgaaWcbaGaemyAaKgabeaakiabg2da9iabcIcaOiabigdaXiabc+caViabcYha8jabdoeadnaaBaaaleaacqWGPbqAaeqaaOGaeiiFaWNaeiykaKYaaabuaeaaieqacqGF4baEaSqaaiab+Hha4jabgIGiolabdoeadnaaBaaameaacqWGPbqAaeqaaaWcbeqdcqGHris5aaaa@4363@

4. Merge clusters *C*_*i *_and *C*_*j *_if *r*(*μ*_*i*_, *μ*_*j*_)<*r*_*thresh*_.

5. Repeat steps 2, 3 and 4 until the number of clusters does not change.

### Computing frequency column clusters from BLOCKS, COG and PFAM

To find frequency column clusters in the BLOCKS database [26], each block from BLOCKS was processed to give a new multiple alignment. This was done to balance the set of sequences in the alignment, so that closely homologous sequences were removed. The processing procedure scanned the sequences in each block from the first sequence to the last. The first sequence from the block was always included in the new multiple alignments. The *i*th (*i *= 2÷ *N*, where *N *is the number of sequences in the block) sequence was included in the new alignment only if it was less than 65% identical to all previously included sequences. Thus, all sequences in the resulted multiple alignment were less than 65% identical with each other. After the extraction procedure, only alignments having at least 15 sequences were considered for to further processing to have enough statistical material. In total, 39253 alignment columns were obtained. The average number of sequences in a multiple alignment was 32. The average protein identity in a multiple alignment was 27%.

**Figure 1:**
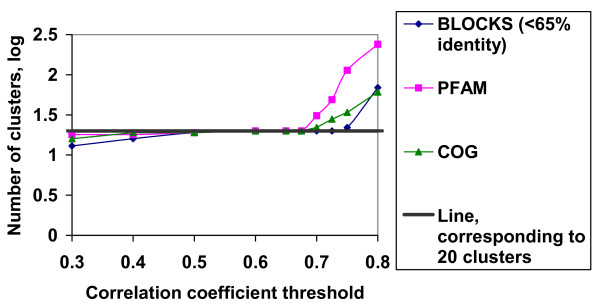
The number of clusters in the BLOCKS (<65% identity), PFAM and COG databases as a function of the correlation coefficient threshold employed in the clustering procedure. Note the horizontal region on the plot when the correlation coefficient threshold is between 0.5 and 0.65.

Fig. 1 shows the number of clusters as dependent on the correlation coefficient threshold r*. The plot shows that when r*< 0.6 the number of clusters is almost constant, whereas after 0.7 the number of clusters sharply increases. In the interval 0.5<r*<0.65, the number of clusters equals 20. Additional file 1 contains 20 average frequency vectors corresponding to each frequency cluster. Each cluster is dominated by a single amino acid, suggesting that columns belonging to one cluster are result from the evolution of just one ancestral amino acid.

To test that this dependence of the number of clusters on the correlation coefficient threshold is the general feature of multiple alignments we applied the same clustering procedure to the COG [27] and PFAM (seed alignments) [28] databases of multiple alignments of protein families. In these cases we did not do any filtering of sequences in multiple alignments but we only weighted sequences using the position-based method of sequence weighing [12]. We obtained the same dependence of the number of clusters on the correlation coefficient threshold with flat regions corresponding to 20 clusters when the correlation coefficient threshold varies from 0.5 to 0.65 and slow decrease when it is less than 0.5 and abrupt rise when it is greater than 0.7 (Fig. 1). Cluster averages were very similar to those shown in Additional file 1 with one dominating amino acid in each cluster, though diagonal values were different reflecting the average identity of protein sequences in multiple alignments of different databases (data not shown).

**Figure 2:**
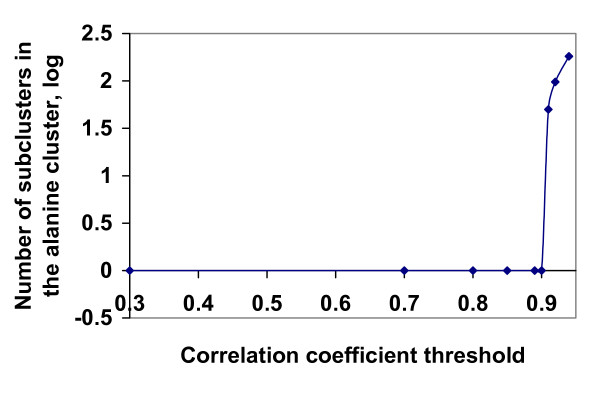
The number of subclusters in the alanine cluster obtained from the BLOCKS database as a function of the correlation coefficient threshold employed in the clustering procedure. Note the sharp rise in the number of subclusters when the correlation coefficient threshold is slightly greater than 0.9.

Any clustering procedure will also attempt to create a hierarchy of clusters. It is interesting to know if there are any other subclusters inside the 20 clusters obtained by our procedure. To answer this question, we applied our procedure to all columns in the BLOCKS database that belong to the alanine cluster. Fig. 2 shows that the alanine cluster does not contain any subtypes, since when the correlation coefficient threshold is less than 0.9 we obtain only one cluster, and when it exceeds 0.9 we have a sharp rise in the number of clusters. Similar curves were obtained for all other 19 clusters (data not shown).

To evaluate the size of clusters on the simplex (Σ*f*_*i *_= 1) in the 20-dimensional space, we generated 1000000 random frequency vectors and counted how many of them fell inside our 20 reference clusters. A frequency vector was considered to be inside a cluster if the correlation coefficient between it and the cluster average vector exceeded 0.5. Only 4% of frequency vectors were inside our 20 clusters, proving the fact that the 20 clusters occupy a negligible part of the total 20-dimensional frequency vector space.

To evaluate how columns in standard multiple alignments are covered by clusters from Additional file 1, we analyzed all 1495235 columns available in the COG database by converting them to frequency vectors and finding the nearest cluster for each column. If the correlation coefficient between the column frequency vector and average the nearest cluster exceeded 0.5, the column was considered to belong to the cluster. Only 23268 columns did not belong to any cluster, less than 2% of the total number of columns.

### Random test

To support the idea of an ancestral residue, the set of artificial alignments was generated in the following way. Parameters of the generation process were chosen to closely mimic parameters of the clustering procedure done on the BLOCKS database. 400 random protein sequences each having the length of 100 amino acids were generated with the standard background frequencies of amino acids. This produced 40000 alignment columns, which is close to the number of alignment columns used in the clustering procedure done on BLOCKS (39253 alignment columns). Each generated sequence was considered to be an ancestral sequence, and the process of column evolution in each column of the generated sequence was modeled by random generation of 32 amino acid residues to obtain the same depth of the generated multiple alignment as the average depth of multiple alignments in our clustering procedure done on BLOCKS. Each of these 32 amino acid residues was generated from its "ancestral" residue with probabilities determined by 4 transition probability matrices [29], corresponding to BLOSUM30 (fast column evolution), BLOSUM50, BLOSUM60 (moderate column evolution), BLOSUM80 (slow column evolution). One of these 4 matrices was randomly assigned to a generated column with the following probabilities 0.42 (BLOSUM30), 0.25 (BLOSUM50), 0.21 (BLOSUM60) and 0.12 (BLOSUM80). These four probabilities were experimentally found values to simulate column evolution as closely as possible as it exists in BLOCKS. The average protein identity in thus generated protein sequences was 32%, which was close to the value obtained in the previous procedure (27%).

The same clustering procedure was applied to randomly generated frequency vectors, and similar 20 clusters were obtained. The Pearson correlation coefficients between corresponding cluster averages from both groups of clusters is >0.99. This proves that the structure of clusters in protein multiple alignments and in the random test is basically the same.

**Figure 3:**
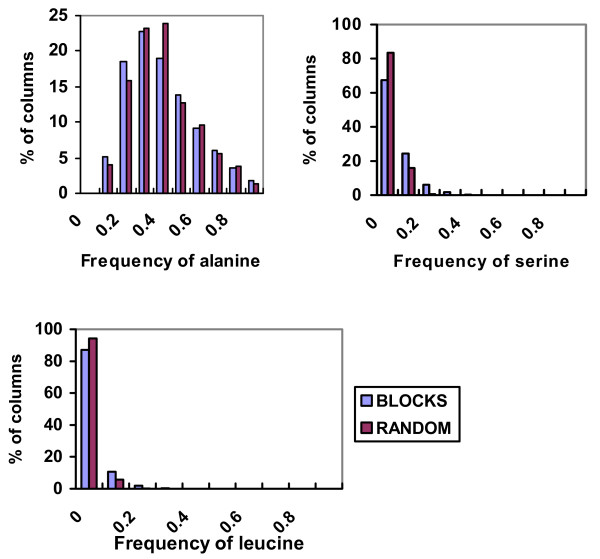
Histograms showing the distributions of the frequency of alanine, serine and leucine in the alanine frequency cluster from the BLOCKS database and from the random protein sequences. a. Frequency of alanine, correlation coefficient between two frequency vectors is 0.986. b. Frequency of serine, correlation coefficient between two frequency vectors is 0.984. c. Frequency of leucine, correlation coefficient between two frequency vectors is 0.998.

Fig. 3 shows the histogram of frequencies of alanine, serine and leucine in the alanine cluster from BLOCKS and from our random simulation. These histograms run pretty close one against each other proving that our random procedure can essentially reproduce the column evolution that exists in nature.

### Assignment of an alignment column to a frequency cluster

To assign columns to clusters, we weight sequences in a multiple alignment to eliminate any distortions in frequency vectors that could be caused by unequal representation of similar sequences in a multiple alignment. Then for all columns frequency vectors are calculated and the correlation coefficient is found between a frequency vector and the nearest cluster average. If this correlation coefficient exceeds a threshold value we assign a column to its nearest cluster, otherwise it is replaced with the "X" symbol.

### How PHOG-BLAST works

To find a similarity score between two multiple alignments, we convert both multiple alignments to "ancestral" sequences as it was described in the previous section. Then our method works in a very similar way as the BLAST [30] and FASTA [31] algorithms.

1. Hash all *l*-tuples from the query "ancestral" sequence to the hash table.

2. Lookup all *l*-tuples from the "ancestral" sequence from the database in the hash table. For each *l*-tuple hit increment the number of hits at a particular diagonal.

3. Leave only diagonals with *N*_*min *_or more *l*-tuple hits for further processing. If no such diagonals are found, declare both compared multiple alignments as non-similar and quit the procedure.

4. At diagonals, extend the leftmost *l*-tuple hit leftward until the total score becomes negative. Memorize the maximum score achieved during this extension and the left end of the segment corresponding to this score. In the same way, extend this *l*-tuple rightward. The result of this extension is a maximal segment pair (MSP). Choose the *l*-tuple which is the left most to the resulting MSP if such *l*-tuple can be found. Apply the same extension process to this *l*-tuple. Scan the diagonal from left to right until no *l*-tuple hits are found anymore. Thus, a diagonal with *N*_*min *_or more *l*-tuple hits can give one or more MSPs. For further processing, leave only MSPs exceeding some diagonal threshold score *SCORE*_*thresh*_. If no such MSPs are found, declare both compared multiple alignments as non-similar and quit the procedure.

5. Order MSPs by their left ends belonging to one member of the pair.

6. Using the following recurrent relationship, obtain the score of the maximal scoring chain of MSPs:

SCORE1=s1SCOREi+1=max⁡j(si+1+SCOREj−G−g⋅max⁡[(lj−ri+1),(mj−pi+1)]:j≤i,lj≥ri+1,mj≥pi+1)
 MathType@MTEF@5@5@+=feaafiart1ev1aaatCvAUfKttLearuWrP9MDH5MBPbIqV92AaeXatLxBI9gBaebbnrfifHhDYfgasaacH8akY=wiFfYdH8Gipec8Eeeu0xXdbba9frFj0=OqFfea0dXdd9vqai=hGuQ8kuc9pgc9s8qqaq=dirpe0xb9q8qiLsFr0=vr0=vr0dc8meaabaqaciaacaGaaeqabaqabeGadaaakeaafaqaaeGabaaabaGaem4uamLaem4qamKaem4ta8KaemOuaiLaemyrau0aaSbaaSqaaiabigdaXaqabaGccqGH9aqpcqWGZbWCdaWgaaWcbaGaeGymaedabeaaaOqaaiabdofatjabdoeadjabd+eapjabdkfasjabdweafnaaBaaaleaacqWGPbqAcqGHRaWkcqaIXaqmaeqaaOGaeyypa0ZaaCbeaeaacyGGTbqBcqGGHbqycqGG4baEaSqaaiabdQgaQbqabaGcdaqadaqaaiabdohaZnaaBaaaleaacqWGPbqAcqGHRaWkcqaIXaqmaeqaaOGaey4kaSIaem4uamLaem4qamKaem4ta8KaemOuaiLaemyrauKaemOAaOMaeyOeI0Iaem4raCKaeyOeI0Iaem4zaCMaeyyXICTagiyBa0MaeiyyaeMaeiiEaGNaei4waSLaeiikaGIaemiBaW2aaSbaaSqaaiabdQgaQbqabaGccqGHsislcqWGYbGCdaWgaaWcbaGaemyAaKMaey4kaSIaeGymaedabeaakiabcMcaPiabcYcaSiabcIcaOiabd2gaTnaaBaaaleaacqWGQbGAaeqaaOGaeyOeI0IaemiCaa3aaSbaaSqaaiabdMgaPjabgUcaRiabigdaXaqabaGccqGGPaqkcqGGDbqxcqGG6aGocqWGQbGAcqGHKjYOcqWGPbqAcqGGSaalcqWGSbaBdaWgaaWcbaGaemOAaOgabeaakiabgwMiZkabdkhaYnaaBaaaleaacqWGPbqAcqGHRaWkcqaIXaqmaeqaaOGaeiilaWIaemyBa02aaSbaaSqaaiabdQgaQbqabaGccqGHLjYScqWGWbaCdaWgaaWcbaGaemyAaKMaey4kaSIaeGymaedabeaaaOGaayjkaiaawMcaaaaaaaa@9248@

where *N*_*msp *_is the total number of MSPs, *s*_*i *_is the score of the *ith *MSP, *l*_*i *_and *r*_*i *_are MSPs' left and right ends in the first sequence, *m*_*i *_and *p*_*i *_are MSPs' left and right ends in the second sequence, *G *is the fixed gap penalty for starting a gap, *g· *max [(*l*_*j*_-*r*_*i*+1_), (*m*_*j*_-*p*_*i*+1_)] is the variable gap extension penalty.

Following the above description of the algorithm, we provide a summary of all parameters that can influence the PHOG-BLAST score between any two multiple alignments:

(i) Profile clusters.

(ii) Substitution matrix between "ancestral" sequences.

(iii) Correlation coefficient threshold r*.

(iv) Tuple size *l*.

(v) Minimum number of tuples on one diagonal *N*_*min*_.

(vi) MSP score threshold *SCORE*_*thresh*_.

(vii) Gap starting penalty *G*.

(viii) Gap continuation penalty *g*.

### Selection of parameters

Extensive computational experiments with PHOG-BLAST allowed us to determine parameter values that provide the best PHOG-BLAST performance. For some parameters, we used traditional values. For other parameters, we had to carry out a number of computational experiments to determine their best values. To determine the best value for a parameter, we varied it over a certain interval, with all other parameter values remaining constant, and monitored the PHOG-BLAST performance.

Profile clusters [see Additional file 1] were used when converting frequency vectors to "ancestral" sequences. We used the well-known BLOSUM62 substitution matrix to compare amino acid residues of "ancestral" sequences.

Our experiments with PHOG-BLAST showed that the correlation coefficient threshold r* that determines whether an alignment column should belong to a cluster is 0.5. It is this correlation coefficient that determines the lower cutoff point on curves showing the dependence of the number clusters on the correlation coefficient threshold employed in the clustering procedure (Fig. 1). Remarkably, it is the same for all three databases – BLOCKS, PFAM and COG, while the upper cutoff point varies in the range from 0.65 to 0.75. With the correlation coefficient threshold 0.5, only less than 2% of alignment columns in BLOCKS, PFAM and COG are out of clusters.

Computational experiments with PHOG-BLAST have also allowed us to determine the best values for other parameters of the algorithm: *N*_*min *_is from 1 to 3, *l *= 2 and *SCORE*_*thresh *_is from 15 to 20. 3-tuples are probably not conserved in protein families from distant organisms, and for *l *= 1 there is a lot of non-homologous diagonals, so it is difficult to choose *N*_*min*_. The conservation of one to three 2-tuples on one diagonal in protein families is an empirical observation that we can use in building the efficient and fast algorithm. For gap penalties, traditional values for them work very well: *G *= 10 and *g *= 1.

## Results

To test the PHOG-BLAST procedure, we compared it with PSI-BLAST [7], a well-known algorithm for fast profile search, and with COMPASS [18], one of the best rigorous profile-profile comparison methods.

The testing procedure was performed on the following databases: COG [27], PFAM [28] and BALIBASE[32]. We have randomly split each multiple alignment from each database into two subalignments and thus obtained two sets of multiple alignments (lets us call them DatabaseA and DatabaseB). Then we applied the two similarity search algorithms to be tested and found BBHs (bi-directional best hits). Ideally, both subalignments that were derived from one multiple alignment should find each other as the BBH. The lower the number of BBHs that are not found, the better the scoring algorithm works.

We used PSI-BLAST in the following way. Using the program formatdb, we made two databases of sequences extracting sequences from the multiple alignments from the databases DatabaseA and DatabaseB. Then for each multiple alignment in DatabaseB we ran the program blastpgp with the option -B against the database of sequences made from DatabaseA. This will find the best sequence in DatabaseA for the profile made from a multiple alignment in DatabaseB. For this best sequence we found a multiple alignment in DatabaseA, to which it belongs, and for this multiple alignment we ran the program blastpgp with the option -B against the database of sequences made from DatabaseB. If the best sequence found in Database B belongs to the multiple alignment, from which we started the matching procedure, then this multiple alignment belongs to the set of BBHs.

Table 1 shows the results of our 3 tests. They show that PHOG-BLAST either has the same performance as PSI-BLAST or is superior to PSI-BLAST in the suggested setting. PHOG-BLAST, however, consumes much less computer resources. It does not need to build and store a 20-dimensional PSSM for each alignment column in the computer memory, a task not realizable on an ordinary personal computer when we need to compare orthologous groups of different taxons.

**Table 1:** This table shows the ability of PHOG-BLAST and PSI-BLAST to match members of different subalignments belonging to one protein family against each other as BBHs when the initial multiple alignment of the protein family was split in two subalignments. See the Results section for explanation

**Test index**	**Database**	**Total number of BBHs to be found**	**Number of BBHs found**	
			
			**PHOG-BLAST**	**PSI-BLAST**
1	COG	3164	3096	3109
2	PFAM	7315	6773	4278
3	BALIBASE	143	70	0

**Figure 4:**
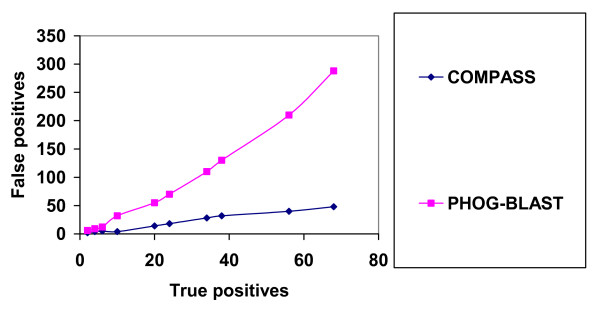
Sensitivity curves of COMPASS and PHOG-BLAST for the remote homology test between PFAM alignments.

Previously developed rigorous profile-profile comparison methods [18-22] use very sophisticated models of profile representation and of course our algorithm can not compete with these methods if it is applied to detect remote homologies. When we used PHOG-BLAST to find BBHs (bi-directional best hits) while building the PHOG database [4], BBHs with scores less than a given threshold (100) were discarded. Such BBHs introduce us into the area of remote homology, and in this area of research the model of profile clusters is too simple to represent the reality. To test how PHOG-BLAST works in the twilight area, we compared PHOG-BLAST with COMPASS. We used COMPASS as it was described in its documentation. From the COMPASS web site [33] we downloaded 1254 PFAM alignments containing at least one sequence from the FSSP database [34]. For each such PFAM alignment we found a FSSP family as it was described in [18]. We used COMPASS and PHOG-BLAST to perform all-against-all comparisons between these 1254 PFAM alignments and to find BBHs. A BBH was considered to be true positive if both PFAM alignments belonged to one FSSP family and false positive if both PFAM alignments belonged to different FSSP families. Fig. 4 shows the sensitivity curves for both methods. Of course, COMPASS showed better performance in the twilight area. However, it took COMPASS 11 hours to perform the test, while PHOG-BLAST required only half an hour of computation time. All tests were performed on 3.2 GHz Pentium 4 computer with 1 GB of RAM running Red Hat Linux 7.3.

To test how COMPASS and PHOG-BLAST work at smaller evolutionary distances (BBH score >100), we used these algorithms to restore protein families in a similar fashion as we did it when compared PHOG-BLAST with PSI-BLAST. To do this test, we have randomly selected 500 PFAM alignments having at least 6 sequences from the earlier mentioned downloaded 1254 PFAM alignments. From each such PFAM alignments, we randomly selected 6 sequences, and from these sequences we made two subalignments each having 3 sequences randomly selected from this group of 6 sequences. We used COMPASS and PHOG-BLAST to find BBHs between these two databases each having 500 alignments consisting of 3 sequences. All tests were performed on 3.2 GHz Pentium 4 computer with 1 GB of RAM running Red Hat Linux 7.3. It took COMPASS 40 minutes to perform the test, while PHOG-BLAST required only 2 minutes of computation time. COMPASS was able to correctly restore 490 BBHs with no false positives, while PHOG-BLAST restored 465 BBHs with no false positives. This observation together with our experience of using PHOG-BLAST when we built the PHOG database [4] demonstrates that PHOG-BLAST used in this setting does not create false positive BBHs and finds 5 % less BBHs than rigorous profile-profile methods.

## Discussion

We developed a new algorithm for finding similarity between protein multiple alignments by finding a way to convert multiple alignments into "ancestral" sequences. The justification for this conversion was our clustering procedure done on frequency distributions and the random simulation of the column evolution. Our algorithm shows performance comparable with PSI-BLAST, or better, but it runs much faster. Our results demonstrate that despite the seeming infiniteness of column space, it is possible to split it into 20 types and to indicate their evolutionary origin. Our "ancestral" sequences are similar to consensus sequences used for representing protein multiple alignments. However, we think that our approach to convert a multiple alignment into a consensus sequence is more precise, since we have a clearer evolutionary base for such conversion. This approach helped us to develop a very precise algorithm for finding similarity between multiple alignments.

In building frequency distributions we did not use pseudo-counts. Any inclusion of pseudo-counts in any form when building frequency distributions only deteriorated the performance of PHOG-BLAST. Since we found 20 condensation points in the column space, this contrasts with the approach taken in [14], where an arbitrary decision was made that the Dirichlet mixture consists of nine Dirichlet densities. This raises an intriguing question: what if we assume that each frequency distribution belongs to one of 20 column clusters modeled as closely as possible as Dirichlet densities? Since mixture coefficients that link Dirichlet densities together reflect the probabilities with which each Dirichlet density occurs in the mixture, these coefficients can be put simply equal to the frequencies of 20 amino acids in protein databases.

Our methodology of testing profile algorithms can also provide a framework for comparing different sequence weighting methods. Within our testing framework, the smaller the number of BBHs that are not found, the better a particular sequence weighting method works.

We have not found any subtypes for profile clusters. However, alignment columns that are descendents of one ancestral amino acid residue differ in the speed of evolution. When we convert columns to their clusters, fast evolving columns and slow evolving columns would acquire the same symbol. We have not so far devised any way to include this additional information into our method.

## Conclusion

Since PHOG-BLAST deals with "ancestral" sequences, and not with profiles, it can be a useful tool for fast comparing multiple alignments of protein families. This task frequently arises in different areas of comparative proteomics and protein evolution. Our accompanying paper [4] shows how we used PHOG-BLAST in the development of the PHOG database and in the automatic reconstruction of orthologs and paralogs from protein complements of different species.

## Availability and requirements

PHOG-BLAST is freely available in Java programming language from  and requires the Java runtime environment. It is also available as an additional file with this manuscript [see Additional file 2]. The PHOG-BLAST software is provided with no guarantee or warranty of any kind. It may be distributed under the terms of the GNU General Public License.

## Authors' contributions

IM did all computation and wrote the manuscript. AM developed the approach for this research, provided overall guidance and revised this manuscript.

## Supplementary Material

Additional File 1 Average frequencies of frequency column clusters obtained from BLOCKS. Dominating amino acids are shown in bold face. Click here for file

Additional File 2 PHOG-BLAST Click here for file
