# Supplementary material for: PHOG-BLAST – a new generation tool for fast similarity search of protein families
Source: BMC Evol Biol. 2006 Jun 22;6:51. doi: 10.1186/1471-2148-6-51 (PMC1522020; doi:10.1186/1471-2148-6-51)
Supplement: Additional File 1 — Average frequencies of frequency column clusters obtained from BLOCKS. Dominating amino acids are shown in bold face. [file 1471-2148-6-51-S1.doc]

|  | **A** | **R** | **N** | **D** | **C** | **Q** | **E** | **G** | **H** | **I** | **L** | **K** | **M** | **F** | **P** | **S** | **T** | **W** | **Y** | **V** |
| --- | --- | --- | --- | --- | --- | --- | --- | --- | --- | --- | --- | --- | --- | --- | --- | --- | --- | --- | --- | --- |
| **1** | **0.486** | 0.019 | 0.016 | 0.014 | 0.022 | 0.017 | 0.023 | 0.062 | 0.008 | 0.03 | 0.038 | 0.023 | 0.015 | 0.015 | 0.016 | 0.081 | 0.041 | 0.003 | 0.011 | 0.059 |
| **2** | 0.035 | **0.509** | 0.028 | 0.018 | 0.006 | 0.038 | 0.032 | 0.021 | 0.022 | 0.019 | 0.033 | 0.094 | 0.011 | 0.012 | 0.013 | 0.035 | 0.029 | 0.006 | 0.015 | 0.023 |
| **3** | 0.033 | 0.033 | **0.476** | 0.062 | 0.007 | 0.029 | 0.033 | 0.043 | 0.029 | 0.015 | 0.021 | 0.042 | 0.008 | 0.011 | 0.014 | 0.067 | 0.043 | 0.003 | 0.015 | 0.017 |
| **4** | 0.032 | 0.022 | 0.069 | **0.522** | 0.004 | 0.026 | 0.081 | 0.03 | 0.018 | 0.011 | 0.016 | 0.035 | 0.006 | 0.008 | 0.016 | 0.048 | 0.028 | 0.003 | 0.009 | 0.015 |
| **5** | 0.039 | 0.011 | 0.014 | 0.01 | **0.702** | 0.008 | 0.01 | 0.019 | 0.007 | 0.014 | 0.024 | 0.009 | 0.008 | 0.014 | 0.01 | 0.033 | 0.025 | 0.004 | 0.01 | 0.03 |
| **6** | 0.041 | 0.054 | 0.036 | 0.029 | 0.004 | **0.435** | 0.069 | 0.02 | 0.024 | 0.02 | 0.038 | 0.065 | 0.019 | 0.012 | 0.012 | 0.042 | 0.034 | 0.005 | 0.013 | 0.028 |
| **7** | 0.055 | 0.039 | 0.032 | 0.086 | 0.004 | 0.057 | **0.427** | 0.023 | 0.015 | 0.017 | 0.026 | 0.066 | 0.009 | 0.009 | 0.02 | 0.045 | 0.033 | 0.003 | 0.011 | 0.024 |
| **8** | 0.053 | 0.018 | 0.033 | 0.025 | 0.007 | 0.013 | 0.02 | **0.657** | 0.009 | 0.011 | 0.015 | 0.024 | 0.006 | 0.01 | 0.013 | 0.043 | 0.019 | 0.003 | 0.007 | 0.016 |
| **9** | 0.025 | 0.026 | 0.04 | 0.021 | 0.005 | 0.034 | 0.021 | 0.018 | **0.582** | 0.014 | 0.024 | 0.025 | 0.007 | 0.027 | 0.011 | 0.028 | 0.018 | 0.008 | 0.049 | 0.016 |
| **10** | 0.033 | 0.011 | 0.008 | 0.007 | 0.009 | 0.008 | 0.01 | 0.011 | 0.006 | **0.437** | 0.138 | 0.012 | 0.031 | 0.04 | 0.008 | 0.016 | 0.026 | 0.005 | 0.016 | 0.169 |
| **11** | 0.036 | 0.016 | 0.009 | 0.008 | 0.01 | 0.013 | 0.014 | 0.013 | 0.008 | 0.103 | **0.5** | 0.015 | 0.049 | 0.052 | 0.009 | 0.019 | 0.023 | 0.007 | 0.019 | 0.077 |
| **12** | 0.045 | 0.113 | 0.038 | 0.028 | 0.005 | 0.051 | 0.057 | 0.023 | 0.019 | 0.022 | 0.035 | **0.401** | 0.011 | 0.01 | 0.017 | 0.044 | 0.038 | 0.003 | 0.012 | 0.027 |
| **13** | 0.045 | 0.015 | 0.014 | 0.008 | 0.01 | 0.026 | 0.015 | 0.013 | 0.008 | 0.064 | 0.139 | 0.013 | **0.45** | 0.039 | 0.008 | 0.024 | 0.029 | 0.006 | 0.016 | 0.06 |
| **14** | 0.027 | 0.013 | 0.009 | 0.008 | 0.008 | 0.009 | 0.011 | 0.012 | 0.012 | 0.046 | 0.092 | 0.011 | 0.023 | **0.528** | 0.009 | 0.019 | 0.02 | 0.021 | 0.08 | 0.04 |
| **15** | 0.055 | 0.025 | 0.018 | 0.027 | 0.005 | 0.02 | 0.034 | 0.025 | 0.011 | 0.019 | 0.03 | 0.036 | 0.007 | 0.013 | **0.559** | 0.045 | 0.03 | 0.004 | 0.01 | 0.029 |
| **16** | 0.087 | 0.023 | 0.044 | 0.034 | 0.014 | 0.023 | 0.029 | 0.051 | 0.015 | 0.017 | 0.024 | 0.03 | 0.011 | 0.014 | 0.02 | **0.436** | 0.087 | 0.004 | 0.014 | 0.025 |
| **17** | 0.053 | 0.024 | 0.032 | 0.022 | 0.013 | 0.022 | 0.024 | 0.022 | 0.012 | 0.03 | 0.035 | 0.03 | 0.014 | 0.014 | 0.013 | 0.107 | **0.47** | 0.003 | 0.011 | 0.05 |
| **18** | 0.025 | 0.018 | 0.012 | 0.011 | 0.006 | 0.01 | 0.014 | 0.015 | 0.014 | 0.026 | 0.046 | 0.015 | 0.016 | 0.067 | 0.011 | 0.022 | 0.016 | **0.579** | 0.052 | 0.024 |
| **19** | 0.024 | 0.018 | 0.017 | 0.01 | 0.007 | 0.012 | 0.015 | 0.014 | 0.033 | 0.026 | 0.045 | 0.017 | 0.013 | 0.12 | 0.01 | 0.022 | 0.019 | 0.026 | **0.521** | 0.031 |
| **20** | 0.061 | 0.014 | 0.009 | 0.008 | 0.016 | 0.01 | 0.015 | 0.013 | 0.007 | 0.145 | 0.088 | 0.017 | 0.021 | 0.026 | 0.011 | 0.023 | 0.045 | 0.004 | 0.015 | **0.453** |
